# Chromatographic analysis of tryptophan metabolites

**DOI:** 10.1002/jssc.201700184

**Published:** 2017-06-26

**Authors:** Ilona Sadok, Andrzej Gamian, Magdalena Maria Staniszewska

**Affiliations:** ^1^ Laboratory of Separation and Spectroscopic Method Applications, Centre for Interdisciplinary Research The John Paul II Catholic University of Lublin Lublin Poland; ^2^ Laboratory of Medical Microbiology Hirszfeld Institute of Immunology and Experimental Therapy Polish Academy of Sciences Wroclaw Poland; ^3^ Department of Medical Biochemistry Wroclaw Medical University Wroclaw Poland

**Keywords:** chromatography, kynurenines, kynurenine pathway, tryptophan metabolites, tissue analysis

## Abstract

The kynurenine pathway generates multiple tryptophan metabolites called collectively kynurenines and leads to formation of the enzyme cofactor nicotinamide adenine dinucleotide. The first step in this pathway is tryptophan degradation, initiated by the rate‐limiting enzymes indoleamine 2,3‐dioxygenase, or tryptophan 2,3‐dioxygenase, depending on the tissue. The balanced kynurenine metabolism, which has been a subject of multiple studies in last decades, plays an important role in several physiological and pathological conditions such as infections, autoimmunity, neurological disorders, cancer, cataracts, as well as pregnancy. Understanding the regulation of tryptophan depletion provide novel diagnostic and treatment opportunities, however it requires reliable methods for quantification of kynurenines in biological samples with complex composition (body fluids, tissues, or cells). Trace concentrations, interference of sample components, and instability of some tryptophan metabolites need to be addressed using analytical methods. The novel separation approaches and optimized extraction protocols help to overcome difficulties in analyzing kynurenines within the complex tissue material. Recent developments in chromatography coupled with mass spectrometry provide new opportunity for quantification of tryptophan and its degradation products in various biological samples. In this review, we present current accomplishments in the chromatographic methodologies proposed for detection of tryptophan metabolites and provide a guide for choosing the optimal approach.

**Abbreviations**
AA: anthranilic acid
CSF: cerebrospinal fluids
CMEK: capillary micellar electrokinetic chromatography
DBD‐F: 4‐N,N‐dimethylaminosulfonyl‐7‐nitro‐2,1,3‐benzoxadiazole
ECNI: electron capture negative ion
EI: electron impact
HCV: hepatitis C virus
HPLC: high pressure liquid chromatography
ED: electrochemical detection
FD: fluorescence detection
IDO: indoleamine 2,3‐dioxygenase
IFN‐γ: interferon‐gamma
IL: interleukin
KAT: kynurenine aminotransferase
Kyn: kynurenine
Kyna: kynurenic acid
NAD+: nicotinamide adenine dinucleotide
ODS column: octadecyl silica column
PIC: picolinic acid
PCA: perchloric acid
(R)‐DBD‐PyNCS: (R)‐4‐(3‐isothiocyanatopyrrolidin‐1‐yl)‐7‐(N,N‐dimethylaminosulfonyl)‐2,1,3‐benzoxadiazole
Quin: quinolinic acid
TCA: trichloric acid
TDO: tryptophan dioxygenase
Trp: tryptophan
XA: xanthurenic acid
3HAA: 3‐hydroxyanthranilic acid
3HKyn: 3‐hydroxykynurenine

## 1 INTRODUCTION

**Figure 1:**
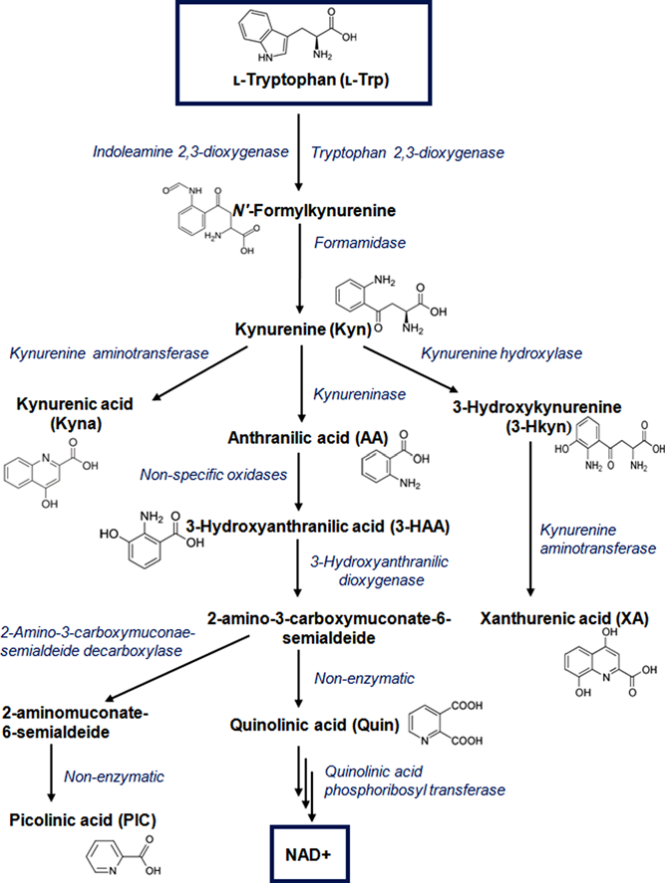
Scheme of ʟ‐tryptophan metabolism via kynurenine pathway

Tryptophan (Trp) is an essential amino acid important for living organism. Less than 1% of dietary Trp is used for protein synthesis, and the rest is degraded through decarboxylation, transamination, hydroxylation, or oxidation 1, leading to generation of physiologically significant compounds such as neuroactive tryptamine, neuroprotective melatonin, or immunosuppressive kynurenine (Kyn). About 80–90% of dietary Trp is metabolized into Kyn by the so‐called kynurenine pathway (Fig. 1) and generation of nicotinamide adenine dinucleotide (NAD), an important enzyme co‐factor. In addition, the methoxyindole pathway utilizes about 1–2% of ʟ‐Trp 2 and provides the neuroactive compounds serotonin and melatonin.

Trp degradation by the kynurenine pathway occurs through several steps and is initiated by activation of two enzymes – extrahepatic indoleamine 2,3‐dioxygenase (IDO; depleting ʟ‐ and ᴅ‐Trp) and hepatic tryptophan 2,3‐dioxygenase (TDO; selective to ʟ ‐Trp) 3, 4. The ratio of kynurenine to tryptophan concentration (Kyn/Trp) reflects IDO and TDO activity and is widely used for monitoring tryptophan metabolism, while the absolute serum Trp level depends on the dietary uptake and is not a reliable parameter 3. Normally, TDO is mainly expressed in the liver and is responsible for regulation of the serum Trp homeostasis. Its expression is induced by corticosteroids 5, however, usually available in liver‐free heme that is necessary for enzymatic activity is sufficient for saturation of only 50% of the available protein.

In contrast IDO is expressed in several mammalian organs, including the endocrine and central nervous systems, placenta, lung, intestine, immune cells, and epididymis 6. The enzyme expression is induced by cytokines, i.e. interferon‐gamma (IFN‐γ), interleukins (IL‐1α, IL‐1β, IL‐6), tumor necrosis factor alpha, and lipopolysaccharide 7, 8, 9, 10 associated with several pathologic conditions such as infection, cancer, as well as with pregnancy 3, 11, 12.

In the first step of kynurenine pathway, ʟ‐Trp is oxidized by IDO or TDO to N′‐formylkynurenine that is rapidly converted to Kyn 13 (Fig. 1). The concentration of this metabolite in plasma, serum, and brain is usually low (micromolar concentrations) 3, 14, 15 and is metabolized by different downstream enzymes within the kynurenine pathway. Kynurenine aminotransferases (KAT I and KAT II) facilitate generation of kynurenic acid (Kyna) by irreversible transamination of Kyn 16, 17. In the brain, Kyna generated by astrocytes 18 occurs at micromolar concentrations, while its level in cerebrospinal fluids (CSF) is much lower (nanomolar concentration) 15. Kyn can however also be a precursor for 3‐hydroxykynurenine (3HKyn) produced by kynurenine hydroxylase, or for anthranilic acid (AA) formed by kynureninase (Fig. 1). Kynurenine hydroxylase is expressed by several cell types including microglia 19, decidual or placental cells 20 and similarly to IDO this enzyme is also upregulated by proinflammatory cytokines 19.

Subsequently, AA is converted to 3‐hydroxyanthranilic acid (3HAA). During 3HAA depletion, the generated 2‐amino‐3‐carboxymuconate‐6‐semialdeide is either metabolized to picolinic acid (PIC) by 2‐amino‐3 carboxymuconate‐semialdehyde decarboxylase 21 or undergoes spontaneous cyclization into Quin (quinolinic acid). This metabolite is required for nicotinic acid (NAD precursor) generation. The concentration of Quin normally present in the nanomolar range in brain and CSF must be kept low, since an increase to 100 nM has been found neurotoxic 22. As a *N*‐methyl‐d‐aspartate receptor agonist, Quin has been associated with several inflammatory and neurological disorders 23, while PIC shows neuroprotective activity 24.

Since kynurenine pathway is a source of several compounds with diverse biological properties, sometimes causing opposite effects, their concentration is highly regulated in vivo. Disturbance in balance between the kynurenines concentrations may lead to negative effects and often indicates pathology. Trp metabolism has been studied for several decades by many scientists across multiple disciplines. The knowledge obtained brings forth new developments on therapeutic strategies to treat infections, chronic inflammation, cancer, reproduction problems, in addition to useful clinical diagnostics.

Selecting an accurate and reliable method for quantification of individual kynurenines, especially in the complex samples such as tissue, present a challenge in research regarding Trp metabolites. The technological developments, especially recent chromatographic methods coupled with MS detectors, come with novel opportunities however they require costly and sophisticated equipment. Kynurenine detection should also be improved by developing standardized extraction protocols, which require laborious method validation. This review summarizes and analyzes suitability of the existing developments in chromatographic methodologies proposed for quantification of Trp and its kynurenine pathway metabolites. It also brings a handy overview and guide on the selection of an appropriate method for sample preparation and quantification of tryptophan metabolites in variety of biological samples.

## 2 CLINICAL SIGNIFICANCE OF ABNORMALITIES IN THE KYNURENINE PATHWAY

Products of the kynurenine pathway and IDO regulation are of a special interest in studies on immune activation. Increase in Trp catabolism has been observed in several autoimmune disorders 25. Patients with primary Sjögren's syndrome have higher serum Kyn concentration as well as [Kyn]/[Trp] ratio when compared to control group without autoimmune symptoms 3. Enhanced IDO‐induced Trp degradation has been also observed in systemic lupus erythematosus 8. In addition, multiple other studies have suggested a possible relationship between autoimmune disorders such as encephalomyelitis 18, rheumatoid arthritis 26, 27, and multiple sclerosis 28 with abnormalities of Trp metabolism.

The enhanced IDO expression is also observed in various infections including hepatitis C virus (HCV) 29, 30 where patients with HCV had lower serum Trp level as compared to controls. Furthermore, HCV‐infected subjects frequently suffered from anxiety and depression‐related symptoms, and their macrophages showed low IDO activity 29. On the other hand, PIC appears to have antiviral properties through the reduction of viral replication and increase of apoptosis of the infected cells 31.

Other reports show an implication of Trp and its oxidative pathway products in pregnancy outcome and describe a decreased Trp concentration during normal pregnancy 32 associated with immune activation 32. It is known that IDO is involved in the formation of maternal immune tolerance toward fetus antigens in early pregnancy 20, however the mechanisms ensuring receptivity of the endometrium are not fully understood. The Trp depletion hypothesis involving a reduction of free Trp access from local tissue microenvironment and suppression of T‐cell proliferation 11, 33, 34, 35, 36 is one of the accepted explanation of the Trp and IDO role in immune regulation 37. On the other hand, the Trp utilization concept points to production of downstream Trp metabolites through kynurenine pathway to achieve immune regulation by immunosuppressive kynurenines accumulation and not simple decrease in Trp availability 1, 11, 37. More research on the role of Trp catabolism must be undertaken to explain the mechanism governing normal and abnormal pregnancy, including preeclampsia 38, miscarriage 36, or postpartum blues 39.

Some of psychiatric/neurologic symptoms have been also found to correlate with Trp metabolites produced through kynurenine pathway in brain, CSF, astrocytes, plasma, and serum. The imbalance between neurodegenerative and neuroprotective Trp metabolites might explain the cause of major depression 40 while the increased Trp degradation is involved in neuropathogenesis of Alzheimerʼs disease 41, 42, 43, Huntington's disease 44, Parkinson's disease 45, brain injury 46, 47, AIDS dementia complex 48, meningitis 49, chronic migraine 50, and amyotrophic lateral sclerosis 51. In patients with schizophrenia an enhancement of TDO expression has been demonstrated 52.

The neurodegenerative disorders affecting older people can be explained by the observation that [Kyn]/[Trp] ratio correlates with the level of neopterin, an immune activation marker 53 the concentration of which in the central nervous system increases with age in women 54. The correlating Quin generation leads to neuronal cells death that has relevance to Alzheimer's disease and other dementias 43, 55.

The importance of maintaining the balance between different kynurenines illustrates antagonizing effect of Kyna that blocks neurotoxic Quin 56, 57. An in vitro study has shown that Kyna reduces the dopaminergic neuronal death caused by 1‐methyl‐4‐phenylpyridiniumthe that is the best‐characterized toxin inducing pathology resembling Parkinson's disease 58. Thus, it has been hypothesized that dysfunction of Kyna production may lead to neurologic disorders 56, 59.

The increased Kyna concentration is associated also with other neurological conditions and was found elevated in Alzheimerʼs dementia (in brain) 15 or in schizophrenia (in CSF) 23. Interestingly, the lower concentrations of Kyna have been recorded in CSF of Huntington's disease 57 and depressive patients 48 compared to control individuals. Important contribution to Kyna level has ᴅ‐amino acid oxidase (DAAO, a susceptibility gene of schizophrenia) converting ᴅ‐Kyn to Kyna 59, 60, 61.

The role of kynurenines in mechanism of neurologic disorders was also shown for 3HKyn that is cytotoxic for cultured neuronal cell 62, 63. This metabolite was found to accumulate in patient's brain 64 and is considered a detrimental key player in Alzheimerʼs 41, Huntington's 44 diseases, and hepatic encephalopathy 65.

Attention is drawn by the significant amount of research on possible role of kynurenine pathway in cancer, since Kyn suppresses anti‐tumor immune response leading to development and progression of malignancy. This phenomenon has been mainly attributed to IDO activity 66, 67, 68, 69, however TDO must also be considered in cancer pathogenesis. Optiz and coworkers 67 confirmed that TDO is the central Trp degrading enzyme responsible for Kyn release in human glioma cells. The evidence supporting IDO/TDO overexpression in cancer 70, 71 presents the therapeutic opportunity for tumor rejection induced by agents inhibiting IDO and/or TDO that could have clinical applications 72, 73, 74.

Finally, there are other conditions where Trp metabolites might play a negative role i.e. cataract 75, 76, 77, 78, UV‐induced skin defects 79, and protective role as eye UV filters 80. In addition, there are reports that link kynurenine pathway metabolites with chronic renal insufficiency 81 and suicidal tendency 82.

## 3 SAMPLE PREPARATION FOR CHROMATOGRAPHIC ANALYSIS OF KYNURENINES

Measurement of Trp and its metabolites is difficult because of their lability, low physiological concentration, and presence of interfering compounds in biological samples. Therefore, some precautions need to be undertaken when performing analysis. Working with samples quickly, at low temperature and protecting from light has been recommended to reduce degradation of Trp and its downstream metabolites to obtain satisfactory results. In case of biological samples such as plasma, serum, or tissues, the protein precipitation before chromatographic analysis must be performed. Proteins in plasma are frequently removed through a pretreatment of sample with acids and by SPE. Deproteinization with perchloric acid (PCA) 14, 44, 61, 83, 84, 85, 86, 87, 88, 89, 90, 91, 92, 93, 94, 95, 96, 97, 98, 99, 100, 101 and trichloroacetic acid (TCA) 29, 54, 101, 102, 103, 104, 105, 106, 107 might be used for this purpose. The sample mixed with acid is shaken and centrifuged to separate precipitated proteins from Trp and kynurenines present in supernatant. However, indole derivatives are sensitive to acidic conditions 99, 108 and precipitation with TCA lowers the Kyn signal 99. Some authors had also proposed the use of other acids like sulfosalicylic acid 95, hydrochloric acid 15, 109, mixture of ascorbic acid, and PCA 26 or TCA with addition of hydrochloric acid 61 or acetonitrile 110. The above agents seem not to be optimal for kynurenines quantification and better choice might be deproteinization with methanol 2, 111, ethanol 112, 113 (chilled or room temperature), and mixture of ammonium acetate in methanol 60, 114, 115, 116 or ammonium acetate in water 117. In some experiments, to prepare brain extracts, acetone has been also used as a precipitation agent 61, 87, 118. Fukushima at al. had reported that extraction efficiency of Kyna with acetone from brain tissue was approximately 1.6‐fold higher than when using 50 mM ammonium acetate in methanol 118. The reprecipitation is also a good practice to purify kynurenines extract 44, 46. Protein precipitation is carried out at room temperature 93, 97, 98, 100, 105 or samples are incubated at −20°C to ensure complete protein removal 2, 111. The subsequent centrifugation is also frequently performed in low temperature (about 4–5°C) to protect indole derivatives from degradation.

Analysis of kynurenines might be performed using samples spiked with internal standard before deproteinization step. It allows for normalization of matrix effects and improves accuracy and reproducibility of the assay 119. There are several compounds used as the internal standards, for determination of Trp and its metabolites, i.e. 3‐nitro‐ʟ‐tyrosie 26, 44, 46, 105, 106, 120, ethyl‐4‐hydroxy‐2‐quinolinecarboxylate 108, theophylline 98, 6‐methyltryptophan 92, ʟ‐tryptophan methyl ester 121, 5‐hydroxytryptamine 122, indoxyl‐sulfate 123, norvaline 40, 7‐aminoheptanoic acid 124, 8‐aminocaprylic acid 125, or dipicolinic acid 46, 126. Many researchers prefer to use the stable isotopically labeled molecules rather than structural analogs especially for bioanalysis employing LC or GC coupled with MS 2, 84, 86, 111, 127, 128, 129, 130. Isotopically‐labeled internal standards have identical chemical properties like the target analyte and minimize problems with stability, recovery, or ionization efficiency issues in comparison to other internal standards. However, the main drawbacks of this approach are high cost and limited availability of the optimal standard 131.

Sample purification by SPE before chromatographic analysis is the useful approach for removing of the interfering compounds present in trace amount. It is based on the nonpolar, polar, ion exchange (cation and anion), and mixed mode interactions of sorbent with an analyte dissolved in liquid phase and subsequent elution with an appropriate solvent. Dowex‐50W cation exchange 15, 84, 109, 132, SepPak 44, 95, and other 41, 102, 106, 108, 116, 120, 129, 133 cartridges have been used for extraction of kynurenines. Implementing of the automated on‐line SPE (using propylsulfonic cartridges) followed by LC–MS/MS shortens the time of analysis reported for quantification of ʟ‐Trp, ʟ‐Kyn, and 3HKyn in human plasma 130. The automated SPE might be also connected with derivatization step of ʟ‐Kyn 115, 116, 120 or Quin to obtain the fluorescent adducts and allow for more sensitive detection 44, 46, 102, 129.

Sample preparation preceding chromatography separation is rather time consuming process, thus methods where sample is directly applied on the column are desired. Kawai's group 134 has described analysis of Kyn and Trp performed by direct injection of filtered plasma (10 μL) into a HPLC system. In this study, the unwanted plasma proteins were removed by trapping on the octadecyl silica precolumn cartridge installed before the main (separation) column 134. This method might generate expected results, but requires frequent replacing of the precolumn due to its heavy wear.

## 4 CHROMATOGRAPHIC ANALYSIS OF TRYPTOPHAN METABOLITES

### 4.1 Separation and detection of kynurenines

#### 4.1.1 Determination of free tryptophan

Measurement of Trp concentration in biological samples is mainly performed with simultaneous detection of its degradation products. LC has been often used for this purpose. There are methods employing various detection modalities: UV absorbance 26, 44, 95, 96, 97, 98, 100, 101, 110, 112, 123, 134, 135, fluorescence 14, 29, 40, 54, 92, 93, 94, 104, 105, 106, 110, 133, 136, 137, electrochemical methods 91, 103, 138 as well as MS 2, 86, 99, 111, 120, 121, 124, 128, 130. The LC separation is mostly achieved on octadecyl silica (ODS) columns. The mobile phases usually contain a small addition of acetonitrile (see Table 1) that shortens a retention time and enhances Trp signal 95. HPLC–UV is an attractive method for clinical applications. However, HPLC–UV methods suffer from low selectivity primarily due to the interference of endogenous compounds present in biological samples 96, 103. The simultaneous determination of Trp and its metabolites using HPLC–UV is hampered by other factors, i.e. amphoteric characteristic of Trp, extremely different concentrations in biological samples (e.g. plasma concentrations of Trp and 3HKyn of healthy subjects is about 50 and <0.13 μM, respectively 130) and relatively long time of analysis 124.

**Table 1:** Chromatographic protocols for ʟ‐Trp quantification

HPLC‐UV					
LOD (μM)	CR (μM)	Mobile phase composition	λ(nm)	Application	Reference
0.069	1.22–97.93	10% (v/v) CH_3_CN in H_2_O, pH adjusted with H_3_PO_4_ to 2.7	273	Human plasma Rat plasma	122
1.32	2.45–146.90	5 mM CH_3_COONa, 8% v/v CH_3_CN	267	Human plasma	112
0.12	3.67–470.00	15 mM CH_3_COONa, 6 % v/v CH_3_CN, pH adjusted with CH_3_COOH to 5.5	302	Human plasma	97
0.20	0.80–500.00	15 mM CH_3_COONa, 5% v/v CH_3_CN	225	Human plasma	100
0.05	2.25–678.00	15 mM acetate buffer (pH 4.0), 5% v/v CH_3_CN	278	Human plasma	101
1.18	2.00–800.00	50 mM phosphate buffer (pH 7.0), 5% v/v CH_3_CN	254	Human plasma	134
0.20	–	100 mM (CH_3_COO)_2_Zn, 50 mM CH_3_COOH, 3% v/v CH_3_CN	250	Human plasma	44, 46
0.02	−	100 mM (CH_3_COO)_2_Zn, 50 mM CH_3_COOH, 3% v/v CH_3_CN	250	Human plasma	26
1.19	3.97–400.00	10 mM acetate buffer (pH 4.5), 6% v/v CH_3_CN	302	Human plasma	98
1.29	5.88–188.00	A: sodium acetate buffer (pH 4.9)/EtOH/H_2_O, B: 100% v/v CH_3_CN, C: 100% v/v H_2_O, D: 1% v/v sodium acetate buffer (pH 5.85)	250	Human plasma	112
0.20	4.90–490.00	15 mM CH_3_COONa, 2.7% v/v CH_3_CN (pH 3.6)	225	Human serum	96
3.50	3.51–225.00	A: 0.1% v/v TCA in H_2_O, B: 0.1% v/v TCA in MeOH	280	Rat serum	110
−	up to 490.00	40 mM acetate/citrate buffer (pH 4.5), 2.5% v/v CH_3_CN	254	Human urine	123
−	−	40 mM CH_3_COONa/citric acid buffer (pH 5), 5% v/v CH_3_CN	280	Dendritic cells	107

HPLC‐FD					
LOD (μM)	CR (μM)	Mobile phase composition	λex/λem (nm)	Application	Reference
0.40	10.00–100.00	20 mM CH_3_COONa, 3 mM (CH_3_COO)_2_Zn, 7% v/v CH_3_CN	344/398	Human plasma	14
−	−	A: Na_2_HPO_4_ in CH_3_CN (pH 6.5), B: 42% v/v H_2_O, 28% v/v CH_3_CN, 32% v/v MeOH	340/440	Human plasma	40
−	0.90 – 26.00	15 mM acetate buffer (pH 4.0), 27 mM CH_3_CN	286/366	Human serum	105
−	0.06 – 222.00	15 mM potassium phosphate buffer (pH 6.4), 2.7 % v/v CH_3_CN	285/365	Human serum	106
0.03	0 – 1000.00	50 mM CH_3_COOH, 250 mM (CH_3_COO)_2_Zn (pH 4.9), 1% v/v CH_3_CN	254/404	Human serum	94
0.001	0.49. – 196.00	50 mM CH_3_COONa, 500 mM (CH_3_COO)_2_Zn, 6% v/v CH_3_CN	254/404	Human serum	93
0.70	6.25 – 100.00	5 mM (CH_3_COO)_2_Zn, 8% (v/v) CH_3_CN, pH adjusted to 4.9	254/404	Human serum	92
0.16	1.00 – 50.00	15 mM phosphate buffer (pH 4.51)	254/404	Human serum	113
0.02	0.05 – 1000.00	30 mM phosphate buffer (pH 8.0), 25% v/v MeOH, tetrabutylammonium hydrogen sulfate	285/360	Human serum	83
−	−	A: 50 mM CH_3_COONa (pH 4.8), B: 50 mM CH_3_COONa (pH 3.65), C: 100% v/v CH_3_CN, D: 100% v/v MeOH	360/350	Human serum	41
0.005	0.05 – 49.00	10% v/v CH_3_CN in H_2_O	285/353	Human serum	137
−	−	100 mM phosphate buffer (pH 3.6), 1 mM EDTA, 5% v/v CH_3_CN	313/420	Human serum Macrophages	30
−	−	40 mM CH_3_COONa/citric acid buffer (pH 5), 5% v/v CH_3_CN	286/366	Dendritic cells	107
0.75	2.50–100.00	A: 15 mM phosphate buffer (pH 6.4), B: 100% v/v CH_3_CN	254/404	Amniotic fluids	136
−	−	0.1 M CH_3_COONH_4_ (pH 4.65)	254/404	Human CFS	54
1.30	−	A: 20 mM CH_3_COONH_4_ in H_2_O (pH adjusted to 5.0 with CH_3_COOH), B: 100% v/v CH_3_CN	285/365	Rat plasma	62
0.005	−	90% v/v H_2_O, 10% v/v CH_3_CN, 0.1% TCA	297/348	Rabbit brain Rabbit amniotic fluids	152

HPLC‐ED					
LOD (μM)	CR (μM)	Mobile phase composition		Application	Reference
0.40	0.80–160.00	0.1 M CH_3_COONa, 0.1 M citric acid, 27 μL EDTA, 20% v/v MeOH, pH adjusted to 5.0 with 5 M NaOH		Human plasma	103
0.24	0.30–100.00	94% v/v 16.2 mM KH_2_PO_4_, 6% v/v CH_3_CN		Human plasma Human cells	138
0.009	0.003–73.40	50 mM sodium phosphate‐acetate buffer (pH 4.1), 10% v/v MeOH, 0.42 mM octanesulphonic acid		Rat brain	91

CMEKC‐ED					
LOD (μM)	CR (μM)	Mobile phase composition		Application	Reference
0.004	0.03 – 10.00	10 M Na_2_HPO_4_‐NaOH buffer (pH 11.0), 40 mM sodium dodecyl sulfate, 3% v/v MeOH		Rabbit urine	143

LC‐MS or LC‐MS/MS					
LOD (μM)	CR (μM)	Mobile phase composition	Monitored ions / transitions (m/z)	Application	Reference
0.003	0.006 – 95.00	A: 2.1% v/v HCOOH (pH 2.0), B: 2.1% v/v HCOOH, 40% v/v CH_3_CN, C: 2.1% v/v HCOOH, 90% v/v CH_3_CN	206>189	Human plasma	99
0.25	6.12–97.93	2% v/v CH_3_CN, 5.2% v/v MeOH, 0.1% HCOOH	204.9>187.9	Human plasma	128
0.40	0.50 – 400.00	A: 650 mM CH_3_COOH, B: 100 mM heptafluorobutyric acid, C: 90% v/v CH_3_CN in H_2_O	206.3>189.1	Human plasma	86
0.03	0.11 – 1200.00	A: 0.2% v/v HCOOH, B: 100% v/v CH_3_CN	205>188>146>118	Human plasma	130
0.018	0.07–1.126	A: HCOONH_4_ in H_2_O (0.05% v/v), pH adjusted to 5.5 with CH_3_COOH, B: 100% v/v CH_3_CN	204.9>188.1>117.8	Rat plasma	108
0.00005	50.00–200.00	10% v/v 10 mM HCO_2_NH_4_ (pH 5.0) in H_2_O, 90% v/v CH_3_CN	556.15>203.25	Human serum	120
0.15	10.00–200.00	A: 80% v/v H_2_O, 20% v/v CH_3_CN, 0.1% v/v CH_3_COOH, B: 20% v/v H_2_O, 80% v/v CH_3_CN, 0.1% v/v CH_3_COOH	556.14	Human serum	124
0.006 0.027	0.015 – 15.00 0.015 – 15.00	A: 0.1% v/v CH_3_COOH, B: 100% v/v MeOH	205>146>188	Human serum Human CFS	2
0.0015	0.0015 – 2.45	A: 0.1% v/v HCOOH in H_2_O, B: 0.1% v/v HCOOH in H_2_O	427	Human urine	140
0.0036	−	A: 28% v/v 0.4 M CH_3_COONH_4_, 32% v/v MeOH, 40% v/v CH_3_CN, B: 32% v/v MeOH, 68% v/v CH_3_CN	383>387>389	Cell culture medium	127
0.01	0.01 – 10.00	A: 5 mM HCOONH_4_, 0.01% v/v TCA, B: 100% v/v MeOH	205>146	Human glioma cells Culture medium	121
−	−	A: 0.1% v/v HCOOH in H_2_O, B: 0.1% v/v HCOOH in CH_3_CN	205.1>118.1	Rat hepatocytes	110
0.01	0.05 – 200.00	A: 0.1% v/v HCOOH in H_2_O, B: 0.1% v/v HCOOH in CH_3_CN	205.1>118.1	Human serum Human urine Cell culture	111
0.049	0.049 – 9.79	A: 20 nM CH_3_COONH_4_, 0.1% v/v HCOOH in H_2_O, B: 100% v/v CH_3_CN	439>170	Rat brain	141

GC‐MS or GC‐MS/MS					
LOD (μM)	CR (μM)		Monitored ions/ transitions (*m/z*)	Application	Reference
0.00042	−		383 > 387 > 389	Cell culture medium	127
−	0.01 – 1.00		608 > 351	Rat brain	119
−	2.50 – 80.00		608	Rat plasma Human plasma	102

LOD, limit of detection; CR, calibration range; λ, wavelength; λex, excitation wavelength; λem,‐ emission wavelength.

LC–ESI‐MS/MS (LC–ESI‐MS) has been proposed to determine ʟ‐Trp and its predominant metabolites of the kynurenine pathway 2, 86, 108, 111, 121, 128. The MS/MS, i.e. LC–MS/MS allows for monitoring many kynurenines and other biologically active compounds such as amino acids, vitamins in single experiment. The small amount of the sample, a short time, and high separation efficiency are the advantages of this method. There are several examples of successful application of LC–MS in analysis of kynurenines, i.e. Möller at al. reported a LC–MS/MS method for simultaneous detection of six different kynurenines (ʟ‐Trp and ʟ‐Kyn, Kyna, AA, 3HAA, Quin) in 10 μL of rat plasma. The target analytes were separated within 10 min analysis 108. Midttun and coworkers demonstrated applicability of LC–MS/MS for determination of 16 compounds including ʟ‐Trp, ʟ‐Kyn, Kyna, 3HKyn, 3HAA, XA (xanthurenic acid), and AA and 13 isotope‐labeled internal standards in human plasma 86. Hényková at al. have proposed a UHPLC–MS/MS protocol for quantitative profiling of ʟ‐Trp, ʟ‐Kyn, Kyna, 3Hkyn, 3HAA, AA, and 11 other tryptophan‐related neuroactive compounds 2 in human serum and CSF within 10 min. Despite impressive developments the main drawback of the LC–MS approaches is a low ionization response of Trp and kynurenines compared to less polar compounds (probably due to their lower surface activity during the electrospray droplet formation), and a significant impact of sample impurities on ionization process 108, 139. Thus, the LC–MS methods require careful sample preparation (i.e. by SPE) that will reduce matrix interference and improve extraction efficiency 108, 140. The addition of internal standards (especially isotope‐labeled analogues) minimizes assay variation. The derivatization step might help in analysis to improve sensitivity and specificity of the method since it increases mass of the target compounds eliminating the interference of matrix components occurring in the low‐*m/z* region 141.

In comparison to LC–MS/MS approaches, the HPLC methods can be also used for simultaneous quantification of a large number of analytes, like it has been done to determine 33 different compounds including ʟ‐Trp and ʟ‐Kyn in human plasma 112. This protocol 112 includes the complex gradient program with four with solvents supplied from 4 different reservoirs, derivatization of analytes and employs two detectors (fluorescence and UV).

The LC separation of target analytes is mostly achieved on octadecyl silica (ODS) columns, however in some reports a triazole‐bonded column has been used. The authors improved sensitivity and specificity of LC–MS/MS assay while working on determination of ʟ‐Trp and ʟ‐Kyn in human serum 120. The method allows ʟ‐Trp and ʟ‐Kyn detection at 50 and 4 pM, respectively. This approach requires derivatization of ʟ‐Trp and ʟ‐Kyn with (R)‐4‐(3‐isothiocyanatopyrrolidin‐1‐yl)‐7‐(*N*,*N*‐dimethylaminosulfonyl)‐2,1,3‐benzoxadiazole, (R)‐DBD‐PyNCS, and purification using SPE. In contrast, an LC–MS protocol employing an ODS column for ʟ‐Trp and ʟ‐Kyn determination in human serum using precolumn derivatization with (R)‐DBD‐PyNCS does not require laborious clean‐up step but it compromises the detection limits of ʟ‐Trp and ʟ‐Kyn to 150 nM 124. The derivatization with dansyl chloride for quantification of ʟ‐Trp and other kynurenines simultaneously with other neuroactive metabolites of dopamine and serotonin metabolic pathway has been also proposed for LC–MS/MS 140, 141. Dansylation and introduction of tertiary amine makes the target compounds easily protonated in positive ESI mode by reducing their polarity. This in consequence increases retention on the reversed‐phase column allowing for better separation of compounds 140.

There are also other chromatographic techniques utilized for determination of ʟ‐Trp (see Table 1). They include capillary GC with negative ion MS 142 or GC with electron capture negative ion MS 119, 127, and capillary micellar electrokinetic capillary chromatography (CMEK) with amperometric detection 143. CMEK is an electrophoretic technique, where the separation is based on the differential migration of the ionic micelles and the bulk running buffer, allowing for higher selectivity.

#### 4.1.2 Kynurenine determination

The most popular method of ʟ‐kynurenine (ʟ‐Kyn) quantification is HPLC with UV detection 14, 26, 29, 40, 41, 44, 54, 82, 90, 92, 94, 96, 97, 98, 101, 105, 106, 112, 113, 123, 134, 135, 136. However, sensitivity 89 and selectivity 96, 103 using HPLC‐based methods may be insufficient for determination of ʟ‐Kyn present at micromolar concentrations in biological fluids or tissues. ʟ‐Kyn quantification using UV detector is performed usually at wavelength of 225 and 360 ± 5 nm (see Table 2). Due to high impact of endogenous compounds observed at 225 nm the favored in clinical analysis is detection at 360 ± 5 nm 41, 92, 98. To improve sensitivity, the fluorescence detectors coupled with HPLC (HPLC–FD) are used for ʟ‐Kyn analysis. This method requires ʟ‐Kyn derivatization and generation of the fluorescent ʟ‐Kyn adduct utilizing the precolumn 115, on‐column 89 or postcolumn 144 methods. The precolumn derivatization of ʟ‐Kyn can be carried out using a benzofurazan‐type reagent: 4‐*N*,*N*‐dimethylaminosulfonyl‐7‐nitro‐2,1,3‐benzoxadiazole (DBD‐F) 115 and generation of the fluorescent adduct. The postcolumn derivatization method developed by Mawatari's group requires a photochemical reaction of ʟ‐Kyn with hydrogen peroxide 144. While the precolumn fluorescence derivatization approach is more complex and time‐consuming, the postcolumn ones require a complicated equipment. Alternatively, the on‐column derivatization techniques seem to be a good choice for a rapid and sensitive ʟ‐Kyn determination, i.e. Lou at al. employed the on‐column fluorescent derivatization of ʟ‐Kyn by zinc acetate 88. This method greatly enhances a fluorescence response of Kyna (see next subsection) and was applied for ʟ‐Kyn determination in human serum. The on‐column derivatization with zinc acetate has been also used for measurement of ʟ‐Kyn and Kyna levels in human serum samples in a single analytical run 89.

**Table 2:** Chromatographic methods for ʟ‐Kyn determination

HPLC‐UV					
LOD (μM)	CR(μM)	Mobile phase composition	λ(nm)	Application	Reference
0.03	1.00 – 10.00	20 mM CH_3_COONa, 3 mM (CH_3_COO)_2_Zn, 7% v/v CH_3_CN	365	Human plasma	14
0.02	0.08 – 50.00	15 mM sodium acetate‐acetic acid, 5% v/v CH_3_CN	225	Human plasma	61
0.014	0.44 – 18.30	15 mM sodium acetate buffer,6 % v/v CH_3_CN, pH adjusted with CH_3_COOH to 5.5	360	Human plasma	97
0.03	0.20 – 21.20	15 mM acetate buffer (pH 4.0), 5 % v/v CH_3_CN	360	Human plasma	101
0.74	1.50 – 600.00	50 mM phosphate buffer (pH 7.0), 5% v/v CH_3_CN	254	Human plasma	134
0.05	−	100 mM (CH_3_COO)_2_Zn, 50 mM CH_3_COOH, 3% v/v CH_3_CN	365	Human plasma	44, 46
–	−	250 mM (CH_3_COO)_2_Zn, 0.9% v/v CH_3_CN, pH adjusted to 5.8 with CH_3_COOH	365	Human plasma	40
0.13	0.42– 20.20	10 mM acetate buffer (pH 4.5), 6% v/v CH_3_CN	302	Human plasma	98
0.05	−	100 mM (CH_3_COO)_2_Zn, 50 mM CH_3_COOH, 3% v/v CH_3_CN	365	Human plasma	26
0.61	1.84– 39.96	A: sodium acetate buffer (pH 4.9)/EtOH /H_2_O, B: 100% CH_3_CN C: 100% H_2_O, D: 1% v/v sodium acetate buffer (pH 5.85)	250	Human plasma	112
−	−	0.1 M CH_3_COOH, 0.1 M CH_3_COONH_4_ (pH 4.65), 2% v/v CH_3_CN	365	Rat plasma	81
−	0.06 – 1.71	15 mM acetic acid‐sodium acetate (pH 4.0), 27 mM CH_3_CN	360	Human serum	105
−	0.09 – 9.84	15 mM potassium phosphate buffer (pH 6.4), 2.7% v/v CH_3_CN	360	Human serum	106
2.00	0.06 – 6.25	5 mM (CH_3_COO)_2_Zn, 8% v/v CH_3_CN (pH adjusted to 4.9)	365	Human serum	92
0.02	0.098 – 49.00	15 mM sodium acetate‐acetic acid, 2.7% v/v CH_3_CN (pH 3.6)	225	Human serum	
0.70	0.25 – 10.00	15 mM phosphate buffer (pH 4.51)	230	Human serum	113
0.10 0.20	0.00 – 100.00	50 mM CH_3_COOH, 250 mM (CH_3_COO)_2_Zn (pH 4.9), 1% v/v CH_3_CN	230 365	Human serum	94
0.10 0.09	0.09 – 4000.00	30 mM phosphate buffer (pH 8.0), 25% v/v MeOH, tetrabutylammonium hydrogen sulfate	265 360	Human serum	83
−	−	A: 50 mM CH_3_COONa (pH 4.8), B: 50 mM CH_3_COONa (pH 3.65), C: 100% v/v CH_3_CN, D: 100% v/v MeOH	365	Human serum	41
−	−	0.1 mM CH_3_COONH_4_, 0.1 M CH_3_COOH, 2% v/v CH_3_CN	365	Human serum Macrophages	12
1.50	0.43 – 28.00	A: 0.1% v/v TCA in H_2_O, B: 0.1% v/v TCA in MeOH	360	Rat serum	110
−	−	40 mM CH_3_COONa/citric acid buffer (pH 5), 5% v/v CH_3_CN	360	Dendritic cells	2
−	up to 480.50	40 mM acetate‐citrate buffer (pH 4.5), 2.5% v/v CH_3_CN	254	Human urine	123
−	−	0.1 M CH_3_COONH_4_ (pH 4.65)	365	Human CFS	54
0.08	0.25 – 50.00	A: 15 mM phosphate buffer (pH 6.4), B: 100% v/v CH_3_CN	230	Amniotic fluids	136

HPLC‐FD					
LOD (μM)	CR (μM)	Mobile phase composition	λex/λem(nm)	Application	Reference
0.04	0.10 – 98.00	250 mM (CH_3_COO)_2_Zn, 50 mM CH_3_COOH, 3% v/v CH_3_CN	365/480	Human serum	88
0.05	0.10 – 19.60	250 mM (CH_3_COO)_2_Zn, 3% v/v CH_3_CN	365/480	Human serum	89
0.50	0.50 – 50.00	A: 95% v/v H_2_O, 5% v/v MeOH, 0.1% HCOOH, B: CH_3_CN/MeOH (95/5), 0.1% HCOOH	553/431	Rat plasma	115
0.02	−	97% v/v 0.05 M Na_2_B_4_O_7_ 0.1 M KH_2_PO_4_ buffer (pH 8.5), 3% v/v EtOH, 60 mM H_2_O_2_	−	Human plasma	144
−	−	250 mM (CH_3_COO)_2_Zn, 50 mM CH_3_COONa, 3% v/v CH_3_CN, pH adjusted to 6.2 with CH_3_COH	365/480	Mouse plasma Mouse brain Mouse liver	61
0.01	−	90% v/v H_2_O, 10% v/v CH_3_CN, 0.1% TCA	364/480	Rabbit brain Rabbit amniotic fluids	152

HPLC‐ED					
LOD (μM)	CR (μM)	Mobile phase composition		Application	Reference
0.50	1.00–80.00	0.1 M CH_3_COONa, 0.1 M citric acid, 27 μL EDTA, 20% v/v MeOH, pH adjusted to 5.0 with 5 M NaOH		Human plasma	103
0.06	0.07–10.00	94% v/v 16.2 mM KH_2_PO_4_, 6% v/v CH_3_CN		Human plasma Human cells	133
0.0062	0.003–72.04	50 mM sodium phosphate‐acetate buffer (pH 4.1), 10% v/v MeOH, 0.42 mM octanesulphonic acid		Rat brain	94

CMEKC‐ED					
LOD (μM)	CR (μM)	Mobile phase composition		Application	Reference
0.0035	0.08–10.00	10 M Na_2_HPO_4_‐NaOH buffer (pH 11.0), 40 mM sodium dodecyl sulfate, 3% v/v MeOH		Rabbit urine	143

LC‐MS or LC‐MS/MS					
LOD (μM)	CR (μM)	Mobile phase composition	Monitored ions / transitions (*m/z*)	Application	Reference
0.0034	0.006–95.00	A: 2.1% v/v HCOOH (pH 2.0), B: 2.1% v/v HCOOH, 40% v/v CH_3_CN, C: 2.1% v/v HCOOH, 90% v/v CH_3_CN	209>192	Human plasma	99
0.024	0.30–4.80	2% v/v CH_3_CN, 5.2% v/v MeOH, 0.1% v/v HCOOH	209>192.1	Human plasma	128
0.007	0.0005–4.00	A: 650 mM CH_3_COOH, B: 100 mM heptafluorobutyric acid, C: 90% v/v CH_3_CN in H_2_O	209.1>174.3	Human plasma	86
0.001	0.05–45.01	A: 0.2% v/v HCOOH, B: 100% v/v CH_3_CN	209>94>192>146	Human plasma	130
0.017	0.07–1.11	A: HCOONH_4_ in H_2_O (0.05% v/v), pH adjusted to 5.5 with CH_3_COOH, B: 100 % v/v CH_3_CN	208.97>192>93.8	Rat plasma	108
0.20	−	A: 20 mM CH_3_COONH_4_ in H_2_O, pH adjust to 5.0 with CH_3_COOH, B: 100% v/v CH_3_CN	209.1	Rat plasma	62
0.00004	1.00 – 5.00	10% v/v 10 mM HCO_2_NH_4_ (pH 5.0) in H_2_O, 90% v/v CH_3_CN	560.15>217.20	Human serum	120
0.15	0.50 – 5.00	A: 80% v/v H_2_O, 20% v/v CH_3_CN, 0.1% v/v CH_3_COOH, B: 20% v/v H_2_O, 80% v/v CH_3_CN, 0.1% v/v CH_3_COOH	560.13	Human serum	124
0.0001 0.00015	0.0008 – 1.50 0.0008 – 1.50	A: 0.1% v/v CH_3_COOH, B: 100% MeOH	209>94>192	Human serum Human CFS	2
0.00002	0.0004 – 2.40	A: 0.1% v/v HCOOH in H_2_O, B: 0.1% v/v HCOOH in H_2_O	442	Human urine	140
0.02	0.02 – 10.00	A: 5 mM HCOONH_4_, 0.01% v/v TCA, B: 100% MeOH	209>146	Human glioma cells Cell culture medium	121
−	−	A: 0.1% v/v HCOOH in H_2_O, B: 0.1% v/v HCOOH in CH_3_CN	209.1>192	Rat hepatocytes	110
0.048	0.05–9.61.00	A: 20 nM CH_3_COONH_4_, 0.1% v/v HCOOH in H_2_O, B: 100% v/v CH_3_CN	442>170	Rat brain	101
0.00004	0.001–7.50	A: 0.1% v/v HCOOH in H_2_O, B: 0.1% v/v HCOOH in CH_3_CN	209.1>192.0	Human serum Human urine Cell culture	111
0.017	−	A: 28% v/v 0.4 M CH_3_COONH_4_, 32% v/v MeOH, 40% v/v CH_3_CN, B: 32% v/v, MeOH, 68% v/v CH_3_CN	383>387>389	Cell culture medium	127

GC‐MS or GC‐MS/MS					
LOD (μM)	CR (μM)		Monitored ions/ transitions (*m/z*)	Application	Reference
0.00055	−		387>391/393>393	Cell culture medium	127
−	0.01 – 1.00		612>442	Rat brain	119
−	1.25 – 40.00		454	Rat plasma Human plasma	102

LOD, limit of detection; CR, calibration range; λ, wavelength; λex, excitation wavelength; λem, emission wavelength.

The LC–MS methods 104, 124 or LC–MS/MS 2, 86, 108, 111, 120, 121, 128, 141 have also been proposed for estimation ʟ‐Kyn levels in biological samples simultaneously with other Trp metabolites. Several of the developed methods for ʟ‐Kyn determination using different chromatographic approaches are compared in Table 2.

Recent studies include exploration and comparison of physiological functions of different enantiomers of Trp and Kyn. The individual or simultaneous ʟ,ᴅ‐Trp and ʟ,ᴅ‐Kyn determination requires a method to separate of chiral compounds. The methods employing precolumn diastereomer derivatization followed by HPLC separation of Kyn and/or Trp enantiomers and their simultaneous fluorescence detection 116, 125, 145 is used for this purpose. Determination of ᴅ‐Kyn in tissues can be also performed with HPLC–FD. This method employs enzymatic oxidation of ᴅ‐Kyn by ᴅ‐amino acid oxidase and subsequent fluorescent detection of the generated Kyna 61, 112, 146, 147.

#### 4.1.3 Determination of kynurenic acid

The most often employed method for Kyna estimation in biological samples such as plasma 14, 26, 40, 87, 114, 148, serum 89, 93, 94, 95, 148, CSF 54, 149, 150, 151, brain 15, 87, 109, 118, 132, 149, 152, heart 132, liver 87, 132 kidney 132, and lenses 153 is HPLC–FD. Due to very low fluorescence generated by Kyna the accurate quantification of nanomolar concentrations in biological samples requires signal improvement, i.e. by specific chelation with zinc ions 14. The postcolumn 14, 114, 117, 118, 149, 150, 151, 154 or on‐column 26, 40, 44, 81, 89, 93, 94, 109, 153 derivatization is utilized to obtain the fluorescent complex of Kyna with Zn^2+^. For this purpose, the Zn‐containing mobile phases with pH around 6.2 are used and allow for generation of their most reliable derivatives 114. It was also observed that an addition of Zn^2+^ could improve the chromatographic separation of Kyna and Trp and cause a some enhancement of Trp fluorescence signal 14. Increase of Kyna fluorescence is achieved by addition of 50 mM ammonium acetate or ammonium formate. They improve ionization of carboxyl group and in consequence increase formation of Kyna‐Zn^2+^ coordination complex 114. In most HPLC–FD methods, authors applied the mobile phases containing from 0.1 to 0.5 M zinc acetate. This high concentration of salt that is weakly soluble in mobile phase may cause particle crystallization thus caution needs to be taken 89, 93. It is recommended to acidify the mobile phase with acetic acid to reduce a risk of column clotting 26, 81, 99, 109, 114, 117, 118, although low pH has negative impact on Kyna‐Zn^2+^ complex formation and reduces its fluorescence 14, 114. Moreover, several endogenous compounds present in a biological sample might also interfere with the complex formation 118. Finally, due to the shorter a retention time of Kyna on ODS column, the mobile phase containing acetonitrile is frequently used as shown in Table 3.

**Table 3:** Chromatographic methods for Kyna determination

HPLC‐UV					
LOD(nM)	CR (μM)	Mobile phase composition	λ (nm)	Application	Reference
−	up to 528.64	40 mM acetate‐citrate buffer (pH 4.5), 2.5% v/v CH_3_CN	254	Human urine	123
−	−	A: 50 mM CH_3_COONa (pH 4.8), B: 50 mM CH_3_COONa (pH 3.65), C: 100% v/v CH_3_CN, D: 100% v/v MeOH	330	Human serum	41

HPLC‐FD					
LOD (μM)	CR (μM)	Mobile phase composition	λex/λem (nm)	Application	Reference
0.9	0.001 – 0.10	20 mM CH_3_COONa, 3 mM (CH_3_COO)_2_Zn, 7% v/v CH_3_CN	344/398	Human plasma	14
0.002	−	100 mM (CH_3_COO)_2_Zn, 50 mM CH_3_COOH, 3% v/v CH_3_CN	344/390	Human plasma	44, 46
−	−	250 mM (CH_3_COO)_2_Zn, 0.9% v/v CH_3_CN (pH adjusted to 5.8 with CH_3_COOH)	334/388	Human plasma	40
0.0005	0.00 – 1.00	50 mM CH_3_COOH, 250 mM (CH_3_COO)_2_Zn (pH 4.9), 1% v/v CH_3_CN	254/404	Human serum	94
0.002	−	100 mM (CH_3_COO)_2_Zn, 50 mM CH_3_COOH, 3% v/v CH_3_CN	344/390	Human plasma	26
0.011	2.62 – 1047.00	250 mM (CH_3_COO)_2_Zn, 3% v/v CH_3_CN	344/404	Human serum	89
0.00005	0.002– 2.09	50 mM CH_3_COONa, 500 mM (CH_3_COO)_2_Zn, 6% v/v CH_3_CN	344/404	Human serum	93
0.00008	−	0.1 CH_3_COO, 5% v/v CH_3_CN	251/398	Human serum	117
−	−	50 mM CH_3_COOH, 50 mM (CH_3_COO)_2_Zn (pH 4.9), 1.2% v/v CH_3_CN	254/404	Rat plasma	81
0.00016	0.025 – 0.20	0.1M CH_3_COOH, 5% v/v CH_3_CN	251/398	Rat plasma	114
0.0017	−	A: 60% v/v 0.5 M (CH_3_COO)_2_Zn 2, B: 40%: v/v 0.1 M CH_3_COONa, 40% v/v CH_3_CN, pH adjusted to 6.3 with CH_3_COOH	344/389	Rat plasma	109
−	−	5 mM CH_3_COONa, 250 mM (CH_3_COO)_2_Zn, 3% v/v CH_3_CN, pH adjusted to 6.2 with CH_3_COOH	344/398	Rat plasma Rat brain Rat liver	87
−	−	250 mM (CH_3_COO)_2_Zn, 50 mM CH_3_COONa, 3% v/v CH_3_CN pH adjusted to 6.2 with CH_3_COOH	344/398	Mouse plasma Mouse brain Mouse liver	61
−	−	250 mM (CH_3_COO)_2_Zn, 50 nM CH_3_COONa, 2.25% v/v CH_3_CN	344/388	Human CFS	54
0.0001	−	90% v/v H_2_O, 10% v/v CH_3_CN, 0.1% TCA	330/390	Rabbit brain Rabbit amniotic fluids	152

HPLC‐ED					
LOD (μM)	CR (μM)	Mobile phase composition		Application	Reference
0.027	0.003– 79.29	50 mM sodium phosphate‐acetate buffer (pH 4.1), 10% v/v MeOH, 0.42 mM octanesulphonic acid		Rat brain	91

LC‐MS/MS					
LOD (μM)	CR (μM)	Mobile phase composition	Monitored ions / transitions (m/z)	Application	Reference
0.0034	0.006 – 95.00	A: 2.1% v/v HCOOH (pH 2.0), B: 2.1% v/v HCOOH, 40% v/v CH_3_CN, C: 2.1% v/v HCOOH, 90% v/v CH_3_CN	206>189	Human plasma	99
0.004	0.0005 – 0.40	A: 650 mM CH_3_COOH, B: 100 mM heptafluorobutyric acid, C: 90% v/v CH_3_CN in H_2_O	190.3>143.9	Human plasma	86
0.0180	0.07 – 1.12	A: HCOONH_4_ in H_2_O (0.05% v/v), pH adjusted to 5.5 with CH_3_COOH, B: 100% v/v CH_3_CN	189.9>143.7>89	Rat plasma	108
0.00020 0.00015	0.0008 – 1.50 0.0003 – 1.50	A: 0.1% v/v CH_3_COOH, B: 100% v/v MeOH	190>144>172	Human serum Human CFS	2
0.0003	0.001 – 5.00	A: 0.1% v/v HCOOH in H_2_O, B: 0.1% v/v HCOOH in CH_3_CN	190.2>144	Human serum Human urine Cell culture	111
0.0095	−	A: 25% v/v 0.4 M CH_3_COONH_4_, 32% v/v MeOH, 40% v/v CH_3_CN, B: 32% v/v MeOH, 68% v/v CH_3_CN	383>387>389	Cell culture medium	127
−	−	A: 0.1% v/v HCOOH in H_2_O, B: 0.1% v/v HCOOH in CH_3_CN	190.2>144	Rat hepatocytes	110
0.012	0.01 – 1.71	A: 20 nM CH_3_COONH_4_, 0.1% v/v HCOOH in H_2_O, B: 100% v/v CH_3_CN	424>170	Rat brain	141

GC‐MS/MS					
LOD (μM)	CR (μM)	Monitored ions / transitions (*m/z*)		Application	Reference
0.0002	−	368>372>374		Cell culture medium	127

LOD, limit of detection; CR, calibration range; λ, wavelength; λex, excitation wavelength; λem, emission wavelength.

It is also known that due to appearance of the carboxyl group in the chemical structure of Kyna its capacity factor (retention properties on a column) under the mobile phase at pH 6.2 is considerably small 149. To resolve this problem and to improve the separation of Kyna from unknown compounds present in biological samples, a postcolumn derivatization employing a column‐switching HPLC system has been proposed 114. The system consists of two ODS columns connected to trapping column (an anion‐exchange column). Kyna separation from interfering compounds is carried out with the first ODS column and an acidic mobile phase. The peak fraction of Kyna is next trapped on the anion‐exchange column and then is introduced into the second ODS column in the optimal mobile phase (pH 7.0) followed by fluorescence detection of Kyna‐Zn^2+^ complex. This column‐switch HPLC system was successfully applied for Kyna determination in 10 μL of a rat plasma. The sample before chromatographic separation was first deproteinated using 50 mM ammonium acetate in methanol. The detection limit of this method was about 0.16 nM. The method proposed by Mitsuhashi et al. after some improvements was applied to quantify Kyna in human serum 117. The detection limit of the improved column‐switching HPLC method was approximately 0.08 nM (4.0 fmol/injection, S/N = 3). Furthermore, the method required only 7.5 μL of human serum demonstrating its clinical usefulness. The same research group investigated the applicability of the column‐switch HPLC approach to rat brain homogenates after a simple pretreatment including deproteinization with acetone 118 and Zhao's group showed simultaneous determination of Kyn, Kyna, and Trp in human plasma 14.

Kyna level in biological samples might be measured also by LC coupled with MS detectors 2, 86, 111, 141. Amirkhani's group proposed a capillary liquid chromatography with ESI‐MS/MS method for simultaneous determination of Trp, Kyn and Kyna in human plasma 99. Finally, Kyna can be also measured as the pentafluorobenzyl derivative using GC–MS 127.

#### 4.1.4 Determination of 3‐hydroxykynurenine

HPLC with electrochemical detection (ED) represents the widely used method for 3HKyn quantification 44, 61, 65, 81, 91, 155. The samples are first separated using different compositions of mobile phase including phosphoric acid, EDTA, triethylamine, heptane sulfonic acid, and addition of small amount of methanol or acetonitrile. Following separation on column, 3HKyn is detected by oxidation at potential in the range of 0.5 to 0.65 V. The obtained electrical current linearly correlates to the concentration of analyte. The electrochemical detection is known for its high sensitivity, however, the main drawback of this approach is lack of reproducibility caused by electrode clogging 141 and loss in selectivity. The last can be improved by optimization of separation conditions and removing the compounds that undergo reduction or oxidation at the same potential as target analyte. The HPLC–ED with chiral column presents also an advantage to separate 3HKyn enantiomers 87 and for this purpose variant of electrochemical detection of 3HKyn in conjunction with CMEK can be employed 143.

3HKyn shows specific absorbance at 365 nm and this property is utilized in its quantification by HPLC–UV 41, 54, 95, 112, 135, 156, while derivatization with p‐toluenesulfonyl chloride gives a fluorescent derivative analyzed by HPLC–FD 156. The other chromatographic methods of 3HKyn determination in different samples such as plasma 86, 108, 130, serum 2, 111, CSF 2, urine 111, 140, 141, cells 110, 111, 121, and culture medium 121 include several protocols of LC–MS/MS as well as GC–MS 119 after sample derivatization with pentafluoropropionic anhydride and 2,2,3,3,3‐pentafluoro‐1‐propanol, respectively. Comparison of different chromatographic protocols for 3HKyn determination is given in Table 4.

**Table 4:** Chromatographic methods for 3HKyn determination

HPLC‐UV					
LOD (μM)	CR (μM)	Mobile phase composition	λ(nm)	Application	Reference
0.10 0.02	0.00 – 100.00	50 mM CH_3_COOH, 250 mM (CH_3_COO)_2_Zn (pH 4.9), 1% v/v CH_3_CN	230 365	Human serum	94
−	up to 446.00	40 mM acetate‐citrate buffer (pH 4.5), 2.5% v/v CH_3_CN	254	Human urine	123
−	−	0.1 M CH_3_COONH_4_ (pH 4.65)	365	Human CFS	54

HPLC‐ED					
LOD (μM)	CR (μM)	Mobile phase composition		Application	Reference
0.03	−	50 mM H_3_PO_4_, 50 mM citric acid, 60 μM EDTA, 8 mM heptane sulfonic acid, 2 mM NaCl, 5% v/v MeOH, pH adjusted to 3.1 with KOH		Human plasma	46
−	−	1.55 v/v CH_3_CN, 0.9% v/v triethylamine, 0.59% H_3_PO_4_, 0.27 M EDTA, 8.9 mM sodium heptanesulfonic acid		Mouse plasma Mouse brain Mouse liver	61
−	−	0.1 M triethylamine, 0.1 M H_3_PO_4_, 0.3 mM EDTA, 8.2 mM heptane‐1‐sulfonic acid sodium salt, 2% v/v CH_3_CN		Rat plasma	81
−	−	1.5% v/v CH_3_CN, 0.9% v/v triethylamine,0.59% v/v H_3_PO_4_, 0.27 mM EDTA, 8.9 mM sodium heptanesulfonic acid		Rat plasma Rat brain Rat liver	87
0.0056	0.003 – 66.90	50 mM sodium phosphate‐acetate buffer (pH 4.1), 10% v/v MeOH, 0.42 mM octanesulphonic acid		Rat brain	91
−	−	0.1 M citrate buffer (pH 3.0), 8% v/v CH_3_CN, 0.4 mM octyl sulfate		Mosquito larval	55

CMEKC‐ED					
LOD (μM)	CR (μM)	Mobile phase composition		Application	Reference
0.0074	0.03 – 10.00	10 M Na_2_HPO_4_‐NaOH buffer (pH 11.0), 40 mM sodium dodecyl sulfate, 3% v/v MeOH		Rabbit urine	143

LC‐MS/MS					
LOD (μM)	CR (μM)	Mobile phase composition	Monitored ions / transitions (*m/z*)	Application	Reference
0.002	0.0005 – 0.40	A: 650 mM CH_3_COOH, B: 100 mM heptafluorobutyric acid, C: 90% v/v CH_3_CN in H_2_O	225.2>208.3	Human plasma	86
0.005	0.02 – 45.00	A: 0.2% v/v HCOOH, B: 100% v/v CH_3_CN	225>110>208>162	Human plasma	130
0.04	0.08 – 1.35	A: 0.05% v/v HCOONH_4_ inH_2_O, pH adjusted to 5.5 with CH_3_COOH, B: 100% v/v CH_3_CN	153.82>135.8>79.8	Rat plasma	108
0.00015 0.00003	0.0003 – 1.50 0.0023 – 1.50	A: 0.1% v/v CH_3_COOH, B: 100% v/v MeOH	225>110>162	Human serum Human CFS	2
0.009	0.009 – 2.23	A: 0.1% v/v HCOOH in H_2_O, B: 0.1% v/v HCOOH in H_2_O	691	Human urine	140
0.1	0.02 – 10.00	A: 5 mM HCOONH_4_, 0.01% v/v TCA, B: 100% v/v MeOH	225>110	Human glioma cells Culture medium	121
0.001	0.002 – 10.00	A: 0.1% v/v HCOOH in H_2_O, B: 0.1% v/v HCOOH in CH_3_CN	225.2>208	Human serum Human urine Cell culture	111
−	−	A: 0.1% v/v HCOOH in H_2_O, B: 0.1% v/v HCOOH in CH_3_CN	225.2>208	Rat hepatocytes	110

GC‐MS/MS					
LOD (μM)	CR (μM)	Monitored ions/transitions (*m/z*)		Application	Reference
−	0.001 – 1.00	630.0>219.0		Rat brain	87

LOD, limit of detection; CR, calibration range; λ, wavelength.

#### 4.1.5 Determination of 3‐Hydroxyanthranilic Acid and Antrahilic Acid

The 3HAA accumulates in brain and different kinds of cells at nanomolar concentrations. To estimate the physiological role of 3HAA, it is assayed simultaneously with other kynurenines, mainly 3HKyn. The reported chromatographic methods for 3HAA determination are summarized in Table 5. The main approach used for this purpose is LC–MS 2, 86, 110, 111, 121, 127, 140 followed by HPLC coupled with fluorescence 40, 85, 94, 157, 158, UV 95, 135, or electrochemical 46, 85, 91 detectors. It is noticeable, that detection limit of 3HAA improved by 70% after addition of 0.01% TCA to the mobile phase 121.

**Table 5:** Chromatographic approaches for 3HAA determination

HPLC‐UV					
LOD (μM)	CR (μM)	Mobile phase composition	λ (nm)	Application	Reference
−	up to 660.00	40 mM acetate‐citrate buffer (pH 4.5), 2.5% v/v CH_3_CN	254	Human urine	123

HPLC‐FD					
LOD (μM)	CR (μM)	Mobile phase composition	λex/λem (nm)	Application	Reference
−	−	250 mM (CH_3_COO)_2_Zn, 0.9% v/v CH_3_CN, pH adjusted to 5.8 with CH_3_COOH	316/420	Human plasma	40
0.001	0.00–10.00	50 mM CH_3_COOH, 250 mM (CH_3_COO)_2_Zn (pH 4.9), 1% v/v CH_3_CN	320/420	Human serum	94
0.0003	up to 0.50	2 mM hexyl sodium sulfate, 1 mM EDTA, 8% v/v MeOH, 48 mM citric acid, 73 mM NaOH (pH 4.65)	311/414	Rat plasma	157
0.0003	up to 0.50	2 mM hexyl sodium sulfate, 1 mM EDTA, 1.5% v/v MeOH, 48 mM citric acid, 73 mM NaOH (pH 4.45)	311/414	Rat brain	157
0.001	0.005–0.05	90% v/v sodium acetate buffer (pH 5.5), 10% v/v MeOH	316/420	Rat brain	85

HPLC‐ED					
LOD (μM)	CR (μM)	Mobile phase composition		Application	Reference
0.030	−	50 mM H_3_PO_4_, 50 mM citric acid, 60 μM EDTA, 8 mM heptane sulfonic acid, 2 mM NaCl, 5% v/v MeOH pH adjusted to 3.1 with KOH		Human plasma	44, 46
0.005	0.004–97.95	50 mM sodium phosphate‐acetate buffer (pH 4.1), 10% v/v MeOH, 0.42 mM octanesulphonic acid		Rat brain	91

LC/MS or LC/MS‐MS					
LOD (μM)	CR(μM)	Mobile phase composition	Monitored ions / transitions (*m/z*)	Application	Reference
0.002	0.0005– 0.40	A: 650 mM CH_3_COOH, B: 100 mM heptafluorobutyric acid. C: 90% v/v CH_3_CN in H_2_O	154.1 > 80.0	Human plasma	86
0.003.00 0.0015	0.0008–1.50 0.0008–1.54	A: 0.1% v/v CH_3_COOH, B: 100% MeOH	154 > 136 > 108	Human serum Human CFS	2
0.1	0.10–10.00	A: 5 mM CH_3_COONH_4_, 0.01% v/v TCA, B: 100% MeOH	154 > 80	Human glioma cells Culture medium	121
0.003	0.005–10.00	A: 0.1% v/v HCOOH in H_2_O, B: 0.1% v/v HCOOH in CH_3_CN	154.2 > 80.0	Human serum Human urine Cell culture	111
0.002	0.002–3.26	A: 0.1% v/v HCOOH in H_2_O, B: 0.1% v/v HCOOH in H_2_O	387	Human urine	140
−	−	A: 0.1% v/v HCOOH in H_2_O, B: 0.1% v/v HCOOH in CH_3_CN	154.2 > 80	Rat hepatocytes	110
0.003	−	A: 28% v/v 0.4 M CH_3_COONH_4_, 32% v/v MeOH, 40% v/v CH_3_CN, B: 32% v/v MeOH, 68% v/v CH_3_CN	383 > 387 > 389	Culture medium	127

LOD, limit of detection; CR, calibration range; λ, wavelength; λex, excitation wavelength; λem, emission wavelength.

Antrahilic acid is also present at nanomolar concentrations in plasma and brain 85, 108 and similar methodology utilizing HPLC–UV 95, 135, fluorescence 85, 110, LC–MS/MS 2, 108, 111, 143, as well as electrochemical 91 detectors are used for AA quantification as the method of choice for several analyses of urine, blood, and CSF. The lowest LOD was, however, achieved using GC with electron capture chemical ionization (ECNI) MS 127. The chromatographic methods proposed for AA quantification are presented in Table 6.

**Table 6:** Chromatographic approaches for AA determination

HPLC‐UV					
LOD (μM)	CR (μM)	Mobile phase composition	λ (nm)	Application	Reference
−	up to 729.18	40 mM acetate‐citrate buffer (pH 4.5), 2.5% v/v CH_3_CN	254	Human urine	123

HPLC‐FD					
LOD (μM)	CR(μM)	Mobile phase composition	λex/λem (nm)	Application	Reference
0.001	0.005 – 0.05	90% v/v sodium acetate buffer (pH 5.5), 10% v/v MeOH	316/420	Rat brain	85
0.015	0.01 – 0.44	A: 0.1% v/v TCA in H_2_O, B: 0.1% v/v TCA in MeOH	339/419	Rat serum	110

HPLC‐ED					
LOD (μM)	CR(μM)	Mobile phase composition		Application	Reference
0.014	0.004 – 109.38	50 mM sodium phosphate‐acetate buffer (pH 4.1), 10% v/v MeOH, 0.42 mM octanesulphonic acid		Rat brain	87

LC/MS‐MS					
LOD (μM)	CR (μM)	Mobile phase composition	Monitored ions/transitions (*m/z*)	Application	Reference
0.007	0.0005 – 0.40	A: 650 mM CH_3_COOH, B: 100 mM heptafluorobutyric acid, C: 90% v/v CH_3_CN in H_2_O	138.1>120.1	Human plasma	86
0.024	0.09 – 1.50	A: (0.05% v/v) CH_3_COONH_4_ in H_2_O, pH adjusted to 5.5 with CH_3_COOH, B: 100 % v/v CH_3_CN	137.86 >119.9>91.9	Rat plasma	108
0.00050 0.00030	0.002 – 1.50 0.002 – 1.50	A: 0.1% v/v CH_3_COOH, B: 100% MeOH	138>120>92	Human serum Human CFS	2
0.0004	0.001 – 1.00	A: 0.1% v/v HCOOH in H_2_O, B: 0.1% v/v HCOOH in CH_3_CN	138.2>120.0	Human serum Human urine Cell culture	111

GC‐MS/MS					
LOD (μM)	CR (μM)		Monitored ions/transitions (*m/z*)	Application	Reference
0.0001	−		136>140>142	Cell culture medium	127

LOD, limit of detection; CR, calibration range; λ, wavelength; λex, excitation wavelength; λem, emission wavelength.

#### 4.1.6 Determination of quinolinic acid

Majority of methods describing quantification if Quin are based on detection with MS methodology. Several protocols utilizing GC for different biological samples, such as rat brain tissues 84, brain microglia cells, astrocytes, neurons from human fetus 159, whole blood and plasma 84 and different kind of cells 122, 159 have been described. Heyes et al. have proposed estimation of Quin by measuring the volatile derivative (dihexafluoroisopropyl ester) by GC–ECNI‐MS 84. In contrast, Nartisin's GC–ECNI‐MS protocol for simultaneous derivatization of trace concentrations of Quin and ʟ‐Trp, ʟ‐Kyn, Kyna, AA, XA, PIC can be used even without preseparation step 127. The method employs lyophilization of aqueous samples in the presence of tetrabutylammonium hydrogen sulfate followed by base‐catalyzed anhydrous pentafluorobenzylation. The pentafluorobenzyl derivatives of ʟ‐Trp and ʟ‐Kyn can be analyzed using GC–ECNI‐MS at impressively small quantities of femtogram amounts 127. In addition, authors have shown that GC–ECNI‐MS is more sensitive than LC/ECNI‐MS for XA and Quin determination. On the other hand, Eckstein and coworker have proposed a GC–MS/MS method with electron capture chemical ionization (GC–ECCI‐MS) for simultaneous monitoring of 43 amino acids and biogenic amines including Trp, Kyn, Kyna, 3HKyn, and XA in CSF 160. The method is based on sample derivatization with 2,2,3,3,3‐pentafluoro‐1‐propanol and pentafluoropropionic anhydride according to protocol described by Watson 161, and was applied after some modification by Notarangeloʼs group (e.g. they used ECNI detection) for determination of Quin and ʟ‐Trp, ʟ‐Kyn, 3HKyn in a sample of rat brain 119 or just Quin in brain, liver tissue, and plasma from mice 61. Moreover, sample derivatization with 2,2,3,3,3‐penta‐fluoro‐1‐propanol and pentafluoropropionic anhydride was used by Sano and coworkers for determination of Quin and other Trp metabolites in human and rat plasma using GC–ECNI‐MS 102. The femtomolar sensitivity was achieved by Smythe and coworkers measuring hexafluoroisopropyl esters on GC–ECNI‐MS for Quin, PIC, and nicotinic acid determination 162. Dobbie et al. have proposed GC electron impact (EI) MS analysis for Quin derivatized to di‐*tert*‐butyldimethylsilyl ester (tBDMS) 163. Ionization by EI‐MS generally results in better fragmentation compared to ECNI‐MS 126. On the other hand, the ECNI‐MS approach does not require prepurification of Quin nor cleaning up of the generated derivative 126 and has been used to study age‐related changes of Quin level in CSF from children and patients with Huntington's disease 44, as well as in blood samples from patients with chronic brain injury 46.

Alternatively, the LC–MS/MS methods have been also proposed for quantification of Quin in variety of biological samples, i.e. plasma 86, 108, serum 111, 129, urine 111, and cultured cells 111, 127. Sample derivatization improves Quin ionization response in the positive ESI mode and might be advantageous for small amount of sample 129. Unfortunately, the SPE is required to eliminate the interfering compounds 108, 129.

#### 4.1.7 Determination of picolinic acid and xanthurenic acid

The PIC detection in serum 41, 83, plasma 108, 162, brain tissues 162, and culture medium 127, 162 have been reported using HPLC–UV, ion‐exchange chromatography 83, LC–MS/MS 108, and GC–ECNI‐MS 127, 162. The small‐volume sample analysis without compromising of sensitivity is achieved by derivatization using pentafluorobenzyl 127 or hexafluoroisopropyl 162 and GC–ECNI‐MS analysis. The fact that PIC can form complexes with proteins and bind to the resin makes HPLC method difficult to use for PIC determination 83, 129. The addition of ion‐pair reagent tetrabutylammonium hydrogen sulfate to a mobile phase has been found to improve the chromatographic elution of PIC 83.

The XA is frequently measured by LC coupled with MS 86, 110, 111, 127, UV 81, 123, or electrochemical 41, 46, 91 detectors. The buffers with pH about 4.6 are used for mobile phase and wavelength ranging from 254 to 338 nm has been applied for detection. The XA measurement can be also performed by GC–MS/MS 127.

### 4.2 Guides for choosing the most suitable chromatographic method for determination of kynurenines

**Figure 2:**
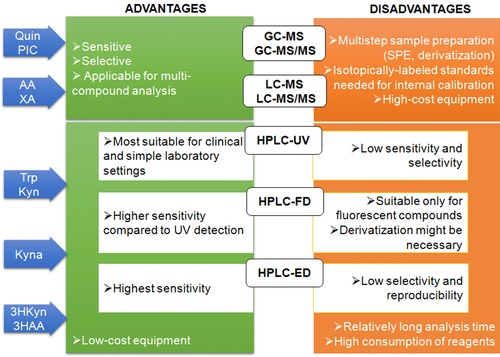
Comparison of chromatographic methods applied for analysis of Trp metabolites

While selecting the method for Trp metabolites assessment at first the expected concentration in the tested specimen should be considered, i.e. Kyn and HAA concentration in human serum is obviously lower (about 1.35 μM and 79 nM, respectively) than that of Trp (about 38 μM) 94. Further, while Trp and Kyn are present in human hippocampus at concentrations about 110 and 20 nM/g tissue, respectively 15, 164, HAA concentration level in rat hippocampus is about 12 fM/g 85. It is also very important to establish, whether the simultaneous analysis of multiple compound is aimed. The most sensitive and selective for this purpose seems to be LC–MS and LC–MS/MS and they are being increasingly used by many researchers for determination of multiple kynurenines. However, these protocols require an expensive equipment, careful sample preparation (including SPE or derivatization) as well as costly isotope‐labeled internal standards. HPLC systems equipped in various types of detectors (UV, fluorescence) are more accessible for analysis in clinical and simple laboratory settings. The HPLC with the UV detector is mainly used for Kyn quantification, although HPLC–UV methods suffer from low sensitivity and selectivity due to an interference of endogenous compounds present in biological samples. The improvement is observed when using HPLC–FD, but the main drawback is that not all kynurenines show fluorescence (e.g. Kyn) or their native fluorescence is too low for accurate quantification without derivatization in complex samples. This methodology is predominantly chosen for Kyna detection. In case of ED detection, the selectivity and repeatability is compromised due to interference of sample components and the electrode clogging, respectively. The HPLC–ED is often used for 3HKyna and 3HAA detection, while the GC–MS or GC–MS/MS is better suited for Quin and PIC due to their low concentration in tissue. These GC assays require expensive equipment and precise sample preparation including purification step and derivatization of target analytes into volatile derivatives. The advantage presented by these methods is high sensitivity and small amount of sample required for analysis. The quick guide for choosing of the optimal approach is summarized in Fig. 2.

## 5 CONCLUDING REMARKS

The involvement of endogenous toxins in the disease mechanism is intensively studied in context of neurodegenerative disorders, cancer, viral infections, or immune regulation. The kynurenine pathway metabolites that include neurotoxic as well as neuroprotective compounds have been widely investigated in this context. Although, a lot of work has been done there is still need to better understand the balance between the level of different Trp metabolites and consequences in organism. These goals cannot be achieved without efficient analytical protocols for accurate and fast determination of kynurenines in body fluids, tissues, and cultured cells. As a result, large number of studies dealing with protocols for kynurenines determination can be found in the literature. Majority of the applied methods regard the chromatographic approach combined with diverse detection modes, including UV, fluorescence, or different types of MS. This diversity of possible methods might be discouraging for many researchers trying to choose the optimal protocol. The issues regarding sample amount, complex composition, fast analytes decomposition, differences in quantity distribution of various kynurenines in the studied sample and amounts present in different tissues should be considered when choosing the chromatographic method.

In future studies aimed to understand the role of Trp metabolites in pathological states, a fast and direct quantification of all kynurenines during a single analytical run would be an ideal solution. This could be achieved with the universal detector allowing for establishing a consistent relationship between the magnitude of response and quantity of target analytes present in the sample. In case of Trp‐derived products LC method is favored, however, as was demonstrated above, no single LC‐coupled detector (UV/visible, fluorescence, electrochemical, mass spectrometric) is capable to detect all kynurenines at physiological level in a given chromatographic eluent. Therefore, future developments in the field of detection technology regarding modern LC are desired. The mass spectrometric detectors are the closest to being called universal detectors of kynurenines. The chromatographic systems coupled with these "near‐universal" detectors can serve as high performance quantification of Trp metabolites by both LC and GC approaches. Moreover, these methodologies require a small injection volume, what is important in case of limited sample amount. On the other hand, these systems are still not included in the basic equipment of laboratories because of their cost and requirement of highly skilled personnel. Therefore, much simpler HPLC systems equipped with UV/visible detectors are mainly utilized. These methods for the multikynurenines analysis unfortunately compromise selectivity and sensitivity. To overcome this problem further improvements in column design and separation conditions should be worked out.

Finally, there is also a need for methods directed at quantification of different enantiomers of Trp and kynurenines. The knowledge on relationship between the ᴅ and ʟ forms of Trp metabolites is important for understanding their involvement in disease mechanism. Currently, this is limited to the first products of the kynurenine pathway (ᴅ‐Trp, ᴅ‐Kyn), thus new methodologies for separation and measurement of chiral compounds must be developed.
